# Melatonin and aggressive behavior: A systematic review of the literature on preclinical and clinical evidence

**DOI:** 10.1111/jpi.12794

**Published:** 2022-03-10

**Authors:** Pasquale Paribello, Mirko Manchia, Marta Bosia, Federica Pinna, Bernardo Carpiniello, Stefano Comai

**Affiliations:** ^1^ Section of Psychiatry, Department of Medical Sciences and Public Health University of Cagliari Cagliari Italy; ^2^ Unit of Clinical Psychiatry University Hospital Agency of Cagliari Cagliari Italy; ^3^ Department of Pharmacology Dalhousie University Halifax Nova Scotia Canada; ^4^ Division of Neuroscience San Raffaele Scientific Institute Milan Italy; ^5^ School of Medicine Vita Salute San Raffaele University Milan Italy; ^6^ Department of Psychiatry McGill University Montreal Quebec Canada; ^7^ Department of Pharmaceutical and Pharmacological Sciences University of Padua Padua Italy; ^8^ Department of Biomedical Sciences University of Padua Padua Italy

**Keywords:** aggressive behavior, fish, humans, melatonin, psychopharmacology, rodents

## Abstract

The melatonin system and circadian disruption have well‐established links with aggressive behaviors; however, the biological underpinnings have not been thoroughly investigated. Here, we aimed at examining the current knowledge regarding the neurobiological and psychopharmacological involvement of the melatonin system in aggressive/violent behaviors. To this end, we performed a systematic review on Embase and Pubmed/MEDLINE of preclinical and clinical evidence linking the melatonin system, melatonin, and melatoninergic drugs with aggressive/violent behaviors. Two blinded raters performed an independent screening of the relevant literature. Overall, this review included 38 papers distributed between clinical and preclinical models. Eleven papers specifically addressed the existing evidence in rodent models, five in fish models, and 21 in humans. The data indicate that depending on the species, model, and timing of administration, melatonin may exert a complex influence on aggressive/violent behaviors. Particularly, the apparent contrasting findings on the link between the melatonin system and aggression/violence (with either increased, no, or decreased effect) shown in preclinical models underscore the need for further research to develop more accurate and fruitful translational models. Likewise, the significant heterogeneity found in the results of clinical studies does not allow yet to draw any firm conclusion on the efficacy of melatonin or melatonergic drugs on aggressive/violent behaviors. However, findings in children and in traits associated with aggressive/violent behavior, including irritability and anger, are emerging and deserve empirical attention given the low toxicity of melatonin and melatonergic drugs.

## 1 INTRODUCTION

Melatonin (*N*‐acetyl‐5‐methoxytryptamine) (MLT) is a neurohormone synthesized in the pineal gland from its precursor serotonin (5‐HT) primarily during the dark phase of the light/dark cycle. In addition, extrapineal synthesis of MLT has been reported in the retina, Harderian gland, and gastrointestinal tract.^1^ MLT regulates several neurophysiological functions, including sleep, mood, circadian rhythm, pain, as well as reproduction, and possesses anti‐inflammatory and antioxidant properties.^2^, ^3^, ^4^, ^5^, ^6^, ^7^ MLT functions are mainly mediated by two seven‐transmembrane G‐protein coupled receptors named MT_1_ and MT_2,_ which differ in terms of molecular structure, chromosomal localization, interactions with different intracellular signaling proteins, and pharmacological affinity to MLT. A detailed description of the differences between these two MLT receptor subtypes lies outside the scope of this systematic review and can be found elsewhere.^3^, ^8^ The presence of MLT receptors in different brain regions as well as in peripheral tissues and organs may account for the different pathophysiological effects of MLT in the human body.^9^

In humans, the onset of MLT secretion is around 21:00–22:00 h, it reaches a peak level of 80–120 pg/ml in the blood between 24:00 and 3:00 h, and the offset of its secretion is in the morning at 7:00–9:00 h, when its serum levels fall down to 10–20 pg/ml.^10^ This circadian production of MLT is under the control of the suprachiasmatic nuclei (SCN), the master clock, in the hypothalamus. The SCN receives input from the retinohypothalamic tract to synchronize with the external light/dark cycle and is connected with the pineal gland through a multisynaptic pathway involving the paraventricular nucleus of the hypothalamus, the sympathetic preganglionic neurons of the intermediolateral cell column in the spinal cord, and the sympathetic noradrenergic neurons of the superior cervical ganglion (SCG).^11^ Once synthesized, MLT has a feedback control on the activity of the SCN by acting on its two receptors, both located at the level of the nucleus.^12^ Growing evidence indicates a strict link between circadian rhythms, behavior, emotions, and mental health/illness.^13^ Consequently, a disruption of the physiological circadian rhythms seems to be implicated in the pathophysiology of psychiatric disorders such as anxiety disorders, major depressive disorder, and bipolar disorder.^14^, ^15^ For instance, there is consistent evidence that the evening chronotype is associated with the severity of psychiatric disorders and may represent a risk factor for their development.^16^ Of note, it has been demonstrated that aggressive behavior tends to be more pronounced in children and adolescents with higher eveningness, for example, those that stay up late at night, and are more mentally and physically active in the late afternoon or evening.^17^ This is of great relevance as aggression is a behavioral disturbance shared by several psychiatric disorders. Despite the well‐established clinical link between circadian disruption and aggression,^18^ a limited number of studies investigated its biological underpinnings (for a review, see Bronsard & Bartolomei^19^). In light of the role of MLT as a zeitgeber for the circadian system, here, we aimed to review current knowledge of the involvement of the MLT system in aggressive/violent behavior by looking at the correlations between changes in MLT levels or MLT receptors and the development of aggressive/violent behavior, and at the possible effects of MLT or MLT receptors ligands in the treatment of aggressive individuals. Importantly, we used a translational approach integrating animal and human findings allowing us to gain novel insights on the role of MLT in the neurobiology and psychopharmacology of aggressive behavior.

### 1.1 Aggressive behavior

The need of increasing the comprehension of the neurobiological underpinnings of aggressive behavior stems from the presence of several unmet goals in the treatment, prevention, and the consequent reduction of the associated socioeconomic burden of this devastating behavioral disturbance. This delay has been determined by several factors. Primarily, the substantial heterogeneity in the phenotypic definition of aggressive behavior decreases the power of studies exploring its biological underpinnings.^20^ This derives mainly, but not exclusively, from the lack of a consensus on the clinical definition (criteria) of aggressive behavior between the different disciplines examining this phenomenon.^21^, ^22^ Indeed, only recently the harmonization of diverse types of measures of aggression has allowed estimating the impact of key environmental components such as the sibling interaction effect.^23^ In addition, the phenotypic heterogeneity has significantly hindered the identification of reliable biomarkers of aggression.^24^ Although multi‐omics approaches are starting to produce promising results,^25^, ^26^ accurate predictive models are still lacking. This is in part a consequence of the complex interaction between genetic susceptibility and environmental factors that determines the biological make‐up of aggression.^27^ The multifaceted neurobiology of aggression involves multiple neurochemical pathways, with certain forms, such as impulsive aggression, possibly associated with dysregulation of the serotonergic signaling system.^22^ However, even for those pathophysiological mechanisms that have been elucidated, there is a poor characterization of causality as well as of the molecular mechanisms leading to treatment efficacy.^22^ Of note, little is known on the role of the MLT system in aggression. Given that phylogenetically MLT is an ancient molecule^28^ and that aggression itself is a primeval ubiquitarian behavioral trait in human and nonhuman vertebrates,^29^ it is conceivable that converging data from clinical and preclinical studies can better inform our knowledge of the biological architecture of aggression. In this context, here, we performed a systematic review of clinical studies and animal models exploring: (1) alterations of the MLT system in aggression and (2) testing whether the perturbation of the MLT system with exogenous MLT administration or MLT receptors ligands can attenuate (or exacerbate) aggression/violence.

## 2 MATERIALS AND METHODS

### 2.1 Search methodology

**Figure 1:**
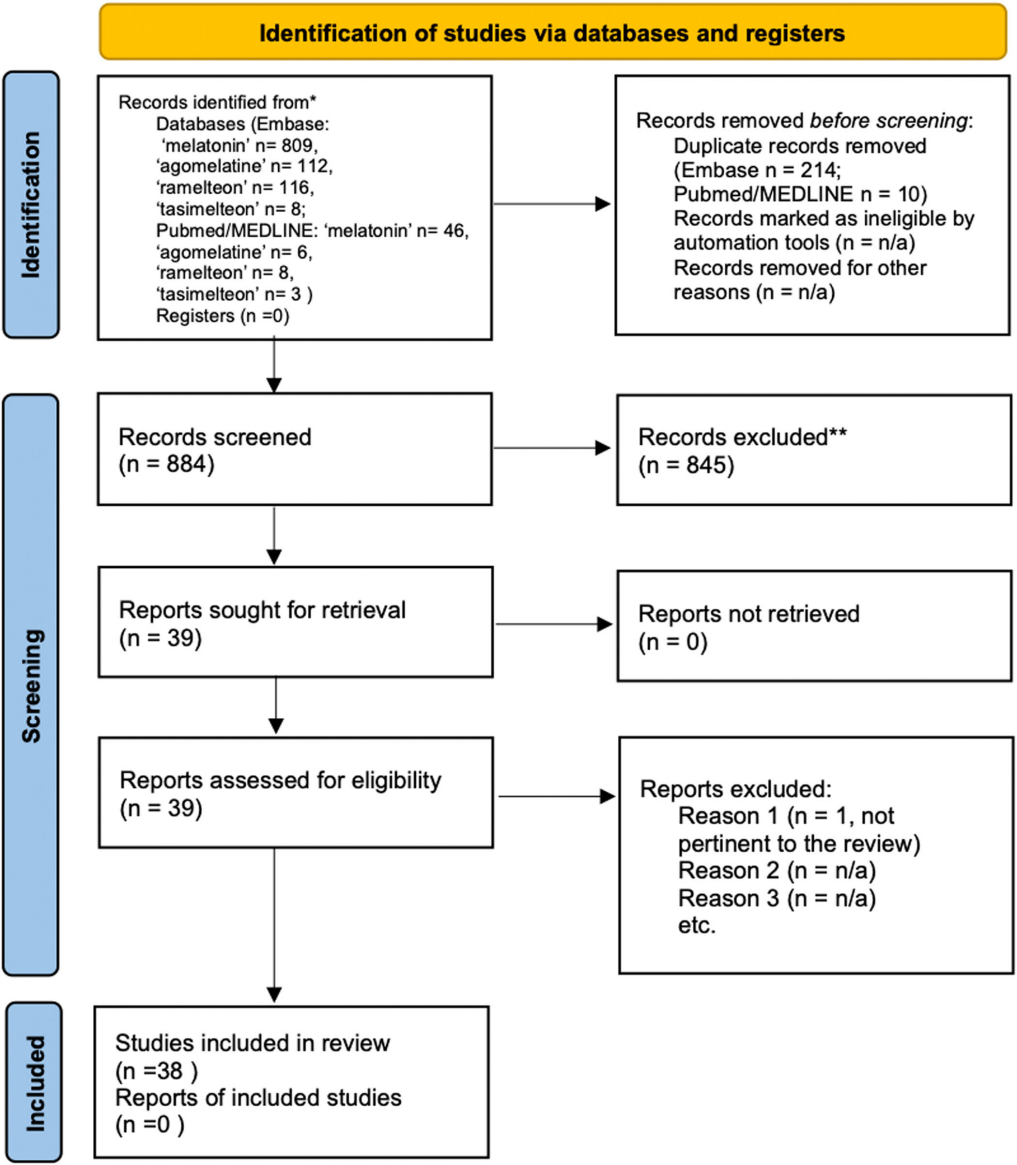
PRISMA 2020 flow diagram for the systematic review

We performed a systematic review in Pubmed/MEDLINE and Embase according to Preferred Reporting Items for Systematic Reviews and Meta‐Analyses (PRISMA) (Figure 1) using the following search terms: “melatonin,” “MT_1_ receptor,” and “MT_2_ receptor” along with “aggression,” “violence,” “impulsivity,” “agitation,” “anger,” and “irritability.” This search strategy was augmented by identifying additional studies replacing the term “melatonin” with “agomelatine,” “ramelteon,” or “tasimelteon” that are all nonselective MT_1_ and MT_2_ receptors agonists approved for clinical use in humans,^30^ as well as with “luzindole” or “4‐phenyl‐2‐propionamidotetraline (4P‐PDOT)” that are a nonselective MT_1_ and MT_2_ receptors antagonist and a selective MT_2_ receptor antagonist, respectively. Further, we examined the references listed in the included papers to identify studies meeting our inclusion and exclusion criteria.

The search was last performed in August 2021, specifically focusing on records regarding trials, case reports, and studies on clinical and preclinical models. Systematic reviews and meta‐analysis were discarded. We first screened titles, and those clearly not in line with the purpose of the review were excluded. Then abstracts were assessed, and lastly, full texts were read, eventually leading to the inclusion or exclusion of the papers, according to the criteria established before the online search. A systematic review of the literature was performed on Medline and EMBASE. The screening of the literature was performed in blind by two investigators (P.P. and S.C.). In the case of disagreement, a third reviewer (M.M.) assessed the paper and a consensus was reached.

### 2.2 Inclusion and exclusion criteria

To be included in the review, research studies had to either: (a) investigate the role of the melatonergic system in aggression in clinical studies or animal models or (b) test the effect of MLT or pharmacological modulators of the MLT receptors and (c) be written in English. We excluded reviews and meta‐analyses of the literature.

### 2.3 Data extraction

The following data considered relevant for the systematic search were extracted from each study and tabulated in a data management software: study, animal species, type of test, the dose of MLT (or treatment), outcome.

### 2.4 Assessment of risk of bias

The risk of bias assessment in randomized controlled trials was performed using the RoB v.2 tool.^31^ Bias domains included in this tool are randomization, deviation from intended interventions, missing data, measurement of outcomes, and selection of reported results. For each tested domain, the relative risk of bias is assigned on a three‐level scale ranging from “Low” to “High” risk of bias. When no information is available to judge a particular item, a “No information” flag is used instead. For each paper, an “overall risk” is also attributed. The risk of bias for nonrandomized studies was evaluated with the ROBINS‐I assessment tool,^32^ an instrument reporting on bias arising from confoundings, selection of study participants, classification of interventions, deviation from intended interventions, missing data, measurement of outcomes, and selection of reported measures. For each tested domain is assigned a score on a four‐level scale ranging from “Low” to “Critical” risk of bias. As previously described for the RoB v. 2, the overall risk of bias is also attributed, and in case of missing data, a “No information” flag is available. A “traffic plot” summary for each test was developed with the web‐based robvis tool.^33^

## 3 RESULTS

Our systematic review identified a total of 38 papers distributed between clinical and preclinical models (Figure 1) showing an association between changes within the MLT system and aggression or an effect of either MLT or compounds targeting MLT receptors on aggression. Eleven papers specifically addressed the existing evidence in murine models, five on fish models, and 21 on humans. We present the main results of these studies in the following sections.

### 3.1 MLT and aggression: Preclinical findings

Preclinical studies examining the effects of MLT administration or changes within the MLT system on aggressive behavior have been conducted in rodents mostly using the resident–intruder paradigm (Table 1), but interestingly, many studies have also been performed in different species of fish, including the cichlid fish and the rainbow trout (Table 2).

**Table 1:** Melatonin, melatonin receptors ligands and aggression: preclinical evidence in rodents

Study	Animal species	Test	Dose of melatonin	Outcome
Demas et al. (2004)^34^	Siberian (adult >60 days) male Hamsters housed on LD photoperiod (16 h light/8 h dark)	Resident–intruder	Exp. 1—15 μg MLT s.c. daily for 10 days, 2 h before the dark phase	Exp. 1—↑ number of attacks and ↓ attack latency
			Exp. 2—15 μg MLT s.c daily for 10 days, 2 h before the dark phase in ADMEDx animals	Exp. 2—No effect of ADMEDx in ↑ number of attacks and ↓ attack latency produced by MLT
			Exp. 3—15 μg MLT s.c daily for 10 days, 2 h before the dark phase in ADx animals	Exp. 3—ADx attenuated ↑ aggression produced by MLT
Fleming et al. (198)^35^	Female golden hamsters housed on LD (14 h light/10 h dark) and SD (10 h light/14 h dark) photoperiods	Agonistic behavior test	Exp. 1—n.a.	Exp. 1—No difference in offensive behaviors between LD and SD; SD ↑ratio offensive to defensive behaviors than LD
			Exp. 2—PNX.	Exp. 2—SD PNX ↓fights than the SD sham
			Exp. 3—25 μg MLT twice a day for 7 weeks in LD animals.	Exp. 3—MLT ↑ offensive behaviors
			Exp. 4—n.a.	Exp. 4—Marginally (*p* = .07) ↑ offensive behaviors in LD OVX

Heinzeller et al. (1988)^37^	Male gerbils housed on LD photoperiod (14 h light/10 h dark)	Resident–intruder	Exp. 1—Evaluation of MLT content before and after a single 3 min encounter in control and SCGX animals	Exp. 1—MLT levels ↑ at the end of the encounter in controls. No change in MLT levels in SCGX animals
			Exp. 2—Evaluation of pineal NAT activity and MLT levels in unstressed animals and in animals after an encounter with a resident (1 min four times a day for a week). Both groups were decapitated before darkness	Exp. 2—MLT levels ↑ with a 2‐h delay in both groups
Jasnow et al. (2002)^36^	Syrian male hamsters (>60 days) housed on long (14 h light/10 h dark)	Resident–intruder	15 μg MLT s.c. daily for 10 days, 2 h before lights out	MLT↑ number of attacks and ↓ attack latency
Laredo et al. (2014)^38^	California male mice (6 months) housed on LD photoperiod (16 h light/8 h dark)	Resident–intruder	0.3 μg/g MLT s.c. daily 3 h before the dark phase for 10 days. One group of animals received also 40 mg/kg luzindole alone or in combination with MLT	↓ attack latency; trend towards ↑ number of bites (*p* = .085). Luzindole did not affect aggression but partially blocked the effects of MLT
Loiseau et al. (2005)^41^	Male Wistar AF rats	Choice behavior in a T‐maze	3 and 10 mg MLT	MLT and agomelatin ↑ number of choices of the large‐but‐delayed reward
			10 and 30 mg agomelatine	
Munley et al. (2020)^45^	Siberian hamster (*Phodopus sungorus*) housed on LD (16 h light/8 h dark) and SD (8 h light/16 h dark)	Resident–intruder paradigm	15 μg/day MLT s.c. injection on a subset of hamsters previously acclimated to LD	Timed MLT in LD animals ↑ aggression to levels similar to those of SD animals
Paterson et al. (1981)^42^	Male mice housed on 10 h light/14 dark cycle	Observation of fight attacks	Daily injection of MLT (100 μg/kg) between 12:00‐13:00 for Days 17–21.	**Single treatments**:
				After ADX ↑attack latency and ↓ attacks (measure with A‐score)
				Treatment with MLT ↓ attacks latencies and ↑ attacks (measure with A‐score)
				**Combined treatments**:
				Both RES + MLT and SO + MLT compares to MLT‐alone tendency to increase aggression (not changes in A‐score). Both RES + MLT and SO + MLT compares to MLT‐alone minor trend toward more attacked and fewer attacking animals. As with ADX and AMG alone the ADX + MLT and the AMG + MLT groups had similar behavioral characteristics. These groups ↓ aggression compares to ADX or AMG alone. ADX + MLT show minimal fighting but not significantly
Rendon et al. (2015)^43^	Siberian (adult >60 days) female hamsters housed on LD (16 h light/8 h dark) and SD (8 h light/16 h dark) photoperiod	Resident–intruder	**In vivo melatonin administration**: 15 μg MLT s.c. daily for 10 days, 2 h before the dark phase in LD female hamsters to examine the influence of melatonin on adrenal steroids and behavior during a period between LD and SD	**In vivo melatonin administration**: Short‐term MLT treatment did not affect reproductive physiology compared with control females.MLT treatment induced shorter latencies to attack and longer attacks
			**In vitro melatonin administration**: modulation of peripheral DHEA release in response to exogenous MLT in adrenals and ovaries across photoperiods. After dissection Paired adrenals were treated with one of four treatment groups: ACTH (0.4 IU), MLT (450 μM), MLT + ACTH (450 μM + 0.4 IU) or a control solution	**In vitro melatonin administration**: Photoperiodic treatment affected reproductive physiology such that SD females had regressed reproductive tissues and displayed elevated levels of aggression.SD females exhibited shorter latencies to first attack, more and longer attacks
Wang et al. (2012)^44^	Male long‐tailed Hamsters housed with a reverse light/dark cycle	Observation of fight attacks	Exp. 1a—Evaluation of testosterone effect on behaviors in LD‐intact males (low MLT, high testosterone) and LD‐castrated males (low MLT, low testosterone)	Exp. 1—Intact males in both photoperiods showed ↑ agonistic behavior than their castrated opponents. LD‐intact males exhibited ↑ aggression and ↓ defensive behavior than LD‐castrated males. SD‐intact males exhibited ↑ aggression and ↓ defensive behavior than SD‐castrated males
			Exp. 1b—Evaluation of testosterone effect on behaviors in SD‐intact males (high MLT high testosterone) and SD‐castrated males (low MLT, low testosterone) and between SD‐castrated males (high MLT, low testosterone)	
			Exp. 2a—Effect of MLT on male dominance under high or low testosterone conditions. The purpose of this experiment was to observe contests between LD‐intact males (low MLT, high testosterone) and SD‐intact males (high MLT, low testosterone)	Exp. 2—In encounters between LD‐ and SD‐intact males ↑win rate against SD intact opponents. No differences in aggression and defensive behaviors were found for pairs of long‐ and SD‐intact males
			Exp. 2b—Effect of testosterone LD‐castrated males (low MLT, low testosterone) and SD‐castrated males (high MLT, low testosterone)	In encounters between LD‐ and SD‐castrated males, SD‐castrated males showed a higher win rate (seven of nine bouts) against LD‐castrated males. No differences in aggression
			Exp. 3a—Effect of implantation of testosterone or MLT on social dominance. Observe contests between intact LD males (low MLT, high testosterone) and intact SD males implanted with testosterone (high MLT, high testosterone)	Exp. 3—LD‐intact males showed a higher win rate against SD‐intact males implanted with testosterone. LD‐castrated males with implanted MLT showed the same win rate against SD‐castrated males
			Exp. 3b—LD‐castrated males implanted MLT (high MLT, low testosterone) and SD‐castrated males (high MLT, low testosterone)	There was no significant difference between LD‐castrated males with implanted MLT and SD‐castrated males for any behavior
Wang et al. (2019)^46^	Male ICR mouse	Resident–intruder	Effects of MLT pretreatment on methamphetamine‐induced aggression (intragastric 2.5, 5, 10 mg/kg MLT 15 min before an intraperitoneal methamphetamine injection 3 mg/kg) compared with two control groups, a methamphetamine‐only group and a vehicle group (normal saline). All chemicals were administered in the first hour of the dark phase, at a maximum dose of 0.1 ml/10 g of bodyweight	The medium MLT dose (5 mg/kg) ↓ number of attacks, total duration of attacks, and ↑ latency to initial attack

Abbreviations: ADMEDx, adrenal demedullations; ADX, adrenalectomies; AMG, aminoglutethimide; DHEA, dehydroepiandrosterone; ICR, Institute of Cancer Research; LD, long‐day photoperiod; MLT, melatonin; n.a., not applicable; OVX, ovariectomy; PNX, pinealectomy; RES, repeated restraint stress; s.c., subcutaneous; SCGX, superior cervical ganglionectomy; SD, short‐day photoperiod; SO, sham operation for adrenalectomy.

**Table 2:** Melatonin (MLT), melatonin receptors ligands and aggression: preclinical evidence in fish

Study	Animal species	Test	Dose of melatonin	Outcome
Audira et al. (2018)^48^	Zebrafish mutants with Leptin a (lepa) gene deficiency	Mirror biting test	12‐fold MLT reduction as compared with controls	Lepa knockout mutants displayed ↓ aggressive behaviors as compared with wild controls
de Amaral et al. (2020)^49^	*Brycon amazonicus* juveniles acclimated for 15 days before the experiment with a 12‐h light period, isolated for 96 h from other animals	Mirror test	Melatonin was diluted directly in the aquarium, before isolating the animal and resulting in three different concentration levels:	Higher frequency of aggressive interaction in the control group as compared with the others; no difference in the frequency of aggressive interactions between the two different MLT concentration levels
			1) Low MLT (1 μmol/L)	
			2) High MLT (10 μmol/L)	
			3) Control (0 μmol/L)	
Larson et al. (2004)^47^	Rainbow trout (1.5 years old) isolated on LD photoperiod in April and May (12 h light/12 h dark).	Observation of fight attacks	Evaluation of MLT and cortisol plasma levels in control, dominant and subordinate individuals during the day and night	Subordinates show ↑ MLT levels than dominants and controls during the night
				Positive correlation between plasma cortisol and MLT in dominant and subordinate animals during the day
Lepage et al. (2005)^50^	Rainbow trout (2 years old) isolated on LD photoperiod (12 h light/12 h dark).	Resident–intruder	Silaning capsule containing MLT in abdominal cavity (1.5% of the body mass)	Exogenous MLT had no direct effect on aggressive behavior excluding the effects of elevated dietary TRP and HPI axis are melatonin mediated
Munro (1986)^51^	Cichlid fish isolated on LD photoperiod (12 h light/12 h dark).	Mirror test conducted 8 h after the start of the light period	Exp. 1—10 μg MLT injected into cranial cavity above the cerebellum	Exp. 1—↓ number of attacks and bites
			Exp. 2—10 μg MLT versus 10 μg MLT + 4 μg naloxone injected into cranial cavity above the cerebellum	Exp. 2—MLT alone or in combination with naloxone equally ↓ number of attacks and bites

#### 3.1.1 MLT and aggression: Preclinical findings in rodents

We identified 11 studies testing the effects of MLT on aggressive behavior in rodents. Demas et al.^34^ studied in the resident‐intruder paradigm the effect of a subcutaneous injection of 15 μg MLT for 10 days, reflecting typical short‐day patterns in Siberian adult male Hamsters housed on long‐day photoperiod. They detected an MLT‐induced decrease in the latency to attack and an increase in the number of attacks to the intruder. Notably, the pro‐aggressive effects of exogenous MLT were attenuated by bilateral adrenalectomies but not by bilateral adrenal demedullations, indicating the involvement of adrenocortical rather than adrenomedullary hormones.^34^ Furthermore, the effect of photoperiod was studied in female Golden Hamsters showing that short‐day animals had a higher ratio of offensive to defensive behaviors than long‐day animals.^35^ Interestingly, this difference was blunted in pinealectomized animals and restored by exogenous injection of MLT, thus demonstrating a pro‐aggressive role for MLT.^35^ These effects were independent of gonadal effects as further confirmed by experiments conducted in ovariectomized hamsters.^35^ Similar findings have also been demonstrated in short‐day male Syrian hamsters, which displayed higher levels of aggressive behavior than long‐day animals, and the injection of MLT in long‐day hamsters significantly increased aggression.^36^ Importantly, these differences in aggressive behavior were not correlated to changes in the levels of testosterone but likely to changes in the HPA axis leading to altered production of adrenal hormones, in particular glucocorticoids.^36^

An interesting experiment by Heinzeller et al.^37^ examined the effects of single and repeated aggressive encounters on the synthesis of MLT in the pineal gland. The authors found an elevation of MLT immediately at the end of the encounter, followed by a decline in the levels of the neurohormone. Conversely, animals with superior cervical ganglionectomy, and thus sympathetic denervated pineal gland, had a fall in MLT levels at the end of the encounter.^37^ Repeated aggressive encounters produced a 2‐h delay in the nocturnal rise of pineal MLT levels.^37^

MLT also increased aggressive behavior in California mice housed under long‐day photoperiod, but interestingly, this pro‐aggressive effect was only partially blocked by the nonselective MT_1_–MT_2_ receptors antagonist luzindole, which per se at the employed dose (40 mg/kg) did not affect aggressive behavior.^38^ In addition, the authors found that these behavioral effects of MLT were not mediated by suppression of estrogen‐dependent genes that were previously associated with increased aggression.^38^

Increased impulsivity is one of the risk factors for aggressive and violent behaviors.^39^, ^40^ Loiseau et al.^41^ found that MLT and agomelatine, a nonselective MT_1_ and MT_2_ receptors agonist and an antagonist to 5‐HT_2B/2C_ receptors, reduced impulsive‐related behavior of Wistar AF rats measured as an increased number of choices of the large‐but‐delayed reward in a T‐Maze apparatus. Interestingly, the effects of MLT on the number of choices were not blocked by the MT_1_ and MT_2_ antagonist S22153, suggesting that the observed effects of MLT on impulsive behavior are not MLT receptors mediated. Paterson et al.^42^ investigated in mice the involvement of the pineal–adrenal axis in relation to territorial aggression. They were able to confirm the enhancing‐fighting properties of MLT in rodents, as well as to demonstrate that these behavioral effects rely on the inhibitory action of this neurohormone on the adrenal cortex.^42^ Indeed, no effects of MLT on aggression were observed in animals with adrenalectomy or treated with aminoglutethimide, a steroid synthesis inhibitor that depletes adrenocortical hormones.^42^ In addition, no effects of MLT were seen in animals undergoing repeated restraint stress and sham‐operated animals for ADX.^42^

Rendon et al.^43^ investigated the link between sex steroids, MLT, and aggressive behavior in female Siberian hamsters, a highly territorial species. They found that short‐day females were more aggressive than long‐day females, and this elevated aggression was concomitant with a dehydroepiandrosterone (DHEA)‐specific increase in adrenal responsiveness. In addition, injection of MLT in long‐day females significantly increased aggressive behavior and circulating levels of DHEA.^43^ Interestingly, the elevation of circulating DHEA induced by MLT is due to increased adrenal and reduced gonadal DHEA release in short‐day females and the opposite in long‐day females. The effect of MLT on agonistic behavior and social dominance and its possible interaction with testosterone was studied in long‐tailed hamsters.^44^ In both short‐ and long‐day photoperiods, intact males displayed more agonistic behavior than castrated males, but no differences were observed between pairs of long‐ and short‐day intact or castrated animals.^44^ Within castrated animals, short‐day males showed a higher win rate against long‐day animals.^44^ Six weeks of MLT implantation increased aggressive and dominant behaviors more in long‐ than in short‐day castrated male hamsters, whereas the implantation of testosterone into short‐day intact males did not affect their subordinate relationship with long‐day intact males. Collectively, Wang et al.^44^ demonstrated that both MLT and testosterone are important for determining social dominance in male hamsters and that MLT is more important during nonbreeding (short‐day) periods while testosterone is during breeding (long‐day) periods. In line with the previous results, Munley et al.^45^ proposed MLT as a possible modulator in the metabolism and synthesis of androgens associated with an aggressive encounter.

MLT also appears to be able of reducing methamphetamine‐induced aggression in the resident–intruder paradigm.^46^ Under the same experimental conditions, the hippocampal levels of dopamine, serotonin, and some of their metabolite levels were influenced by MLT exposure.

#### 3.1.2 MLT and aggression: Preclinical findings in fish

Larson et al.^47^ investigated in rainbow trouts, a species displaying strong dominance hierarchy, whether circulating levels of MLT were associated with the dominance‐subordination relationship. Subordinate fishes displayed increased night‐time levels of MLT, and in both dominant and subordinate fishes, a positive correlation between MLT and cortisol plasma levels was observed during the day but not during the night.^47^ Audira et al.^48^ reported a 12‐fold reduction in MLT levels in Zebrafish leptin knockout mutant. Circadian rhythm appeared significantly altered as compared with controls, with aggressive behaviors also appearing altered: considering the setting and the possible confoundings, more data might be required to better establish a clearer causal link. MLT exposure appeared associated with a reduction in aggressiveness and lipid peroxidation in *Brycon amazonicus*, a species known for engaging in cannibalistic behaviors and for being subject to oxidative stress related to being kept in artificial environments.^49^ As feeding fish with a tryptophan‐enriched diet was known to decrease aggressive behavior, Lepage et al.^50^ investigated whether this effect was mediated by the central serotonergic and/or MLT systems of which tryptophan is the common precursor. Tryptophan‐enriched diet produced a suppression of aggressive behavior and an increase of plasma MLT levels. Conversely, the exogenous administration of MLT producing a similar elevation of plasma MLT levels was ineffective in suppressing aggressive behavior. Citalopram, a serotonin re‐uptake inhibitor, instead induced an aggression‐suppressive effect similar to that produced by the tryptophan‐enriched diet, indicating that the effects of the amino acid were likely mediated by serotonin and not by MLT.^50^ Munro^51^ instead showed in another fish, the *Aequidens pulcher*, that MLT reduced aggression similar to 5‐HT, and that the anti‐aggressive effects of 5‐HT were likely mediated by its conversion into MLT. Indeed, simultaneous treatment with 5‐HT and S‐adenosyl homocysteine, an inhibitor of the conversion of 5‐HT into MLT, blocked the inhibitory effects of 5‐HT on aggression.^51^

### 3.2 MLT and aggression: Clinical findings

MLT and melatonergic agonists have been evaluated in diverse clinical settings as summarized in Table 3. Although case‐report studies might be of limited scientific validity and may lead to over‐interpretation of the findings, we decided to include them as this is the first systematic work examining the link between MLT and aggression. Thus, it may be used to generate new hypotheses for future studies. Asano et al.^52^, ^53^ reported two case reports suggesting the possible efficacy of ramelteon in the management of aggressivity. In the first one, ramelteon significantly improved agitation/aggression and irritability in a 79‐year‐old man patient affected by Alzheimer's disease (AD) with behavioral and psychological symptoms of dementia. The second case report described an improvement in irritable and violent behaviors with ramelteon in a 16‐year‐old boy with high‐functioning autistic disorder diagnosed at the age of 5 years. Parallel to this decrease in impulsive/aggressive behaviors, in both cases, an improvement of sleep was observed (i.e., a reduction of the latency to fall asleep and an increase of the sleep duration). An open‐label study involving individuals affected by Smith‐Magenis syndrome found that tasimelteon resulted in an improvement in parent‐rated behavior and sleep quality as compared to baseline.^54^ In contrast, in a double‐blind randomized placebo‐controlled study of 41 patients with severe AD, MLT did not induce any improvement in neither sleep nor agitation.^55^ Several papers reported on MLT's impact on the risk of postoperative delirium. In a randomized controlled trial involving 148 children (36–37 children per tested dose of MLT) undergoing anesthesia, premedication with MLT reduced in a dose‐dependent fashion the incidence of delirium.^56^ Similar findings were also obtained by Ozcengiz et al.^57^ in a placebo‐controlled, double‐blinded trial in 100 children and by Samarkandi et al.,^58^ who found that premedication with MLT in children undergoing general anesthesia for minor elective surgery reduced or prevented postoperative excitement. Of note, this reduced postoperative excitement was greater with MLT also compared to the benzodiazepine midazolam, indicating that the effect of MLT was not subsequent to increased sedation of the children. In a more recent work, MLT reduced the incidence of sevoflurane‐induced agitation among 120 children (30 children per group) undergoing adenotonsillectomy.^59^ On the contrary, Jaiswal et al. did not find any effect for ramelteon in the prevention of postoperative delirium among individuals undergoing elective pulmonary thromboendoarterectomy.^60^ Similarly, no effect was reported for ramelteon in the incidence of postoperative delirium in a further randomized, placebo‐controlled trial among pediatric individuals undergoing tonsillectomy.^61^ A retrospective, observational study investigated the preventive effect of MLT, ramelteon, or placebo in the incidence of delirium among individuals admitted to a single‐center intensive care unit (ICU). No difference was reported among the included groups, with greater agitation and sedation observed in the MLT cohort. Considering the type of study, no causality link could be established for the observed results.^62^ A double‐blind, randomized placebo‐controlled trial among critically ill patients assessed the effect of MLT in preventing delirium. The patients were randomized 1:1 to receive, at 8 p.m. and at midnight, MLT (3 + 3 mg) or placebo, from the third Intensive Care Unit ICU day until ICU discharge. The authors reported a lower amount of enteral hydroxyzine in the MLT arm, along with an improvement of other neurological indicators (i.e., amount of employed neuroactive drugs, pain, agitation, anxiety, sleep observed by nurses, need for restraints, need for extra sedation, nurse evaluation of sedation adequacy).^63^ A retrospective study^64^ evaluated the efficacy of agomelatine in the management of Behavioral and Psychological Symptoms in Dementia among 75 individuals treated during a 2.5 year period. The included cases had different underlying conditions, including AD, Lewy body, vascular and mixed type dementia. The overall sample presented a significant improvement in the neuropsychiatric scores, specifically in the depression, agitation, irritability, sleep, motor disturbance, and behavior section, but with no observable change for the elation/euphoria symptoms.

**Table 3:** Melatonin, melatonin receptors ligands and aggression: clinical evidence

Study	Disease	Type of study	Patients	Measure of aggression	Dose	Outcome
Asano et al. (2013)^53^	AD	Case report	79‐year‐old man	NPI, BEHAVE‐AD‐FW	8 mg oral ramelteon	Ramelteon ↓ agitation/aggression and irritability subscales
Asano et al. (2014)^52^	ASD	Case report	16‐year‐old boy	Parental report	8 mg oral ramelteon	Ramelteon administration ↓ aggressive episodes
Gehrman et al. (2009)^55^	AD	10‐days double‐blind randomized placebo‐controlled trial with a 5‐day follow‐up	41 patients (61–95 years)	ABRS and CMAI	8.5 mg MTL immediate release	No effect of MLT on agitation compared to placebo
					MLT 1.5 mg sustained release	
Hull et al. (2018)^54^	Smith‐Magenis syndrome	9 to 36 weeks, uncontrolled, before–after, open‐label trial	12 patients (16–38 years)	ABC‐C	20 mg tasimelteon	Reduced levels of ABC‐C total score, and of the Irritability/Agitation/Crying subscale as compared with the baseline
Jaiswal et al. (2021)^60^	Individuals undergoing elective pulmonary thrombo‐endarterectomy	Double‐blind randomized placebo‐controlled trial	120 adult patients	CAM‐ICU	8 mg ramelteon	No difference in the amount of antipsychotic used as compared with placebo; no difference in the CAM‐ICU was reported
Kain et al. (2009)^56^	Surgery with general anesthesia	Double‐blind randomized placebo‐controlled trial	148 children (2–8 years)	mYPAS Impulsivity Scale	MTL (0.05‐0.40 mg/kg)	MTL ↓ the incidence of emergence delirium after general anesthesia
Khalifa et al. (2013)^59^	Prevention of emergence agitation after general anesthesia with sevoflurane for tonsillectomy	Double‐blind randomized placebo‐controlled trial	120 children (3–6 years)	EAS	0.1 mg/kg oral MTL + 15 mg/kg paracetamol	MTL ↓ EA after sevoflurane anesthesia
Komazaki et al. (2020)^61^	Prevention of emergence agitation after general anesthesia with sevoflurane for tonsillectomy	Double‐blind randomized placebo‐controlled trial	48 children (18–119 months)	PAED	0.1 mg/kg oral ramelteon in 5 ml lactose‐containing sirup	No difference in the incidence of emergence agitation
				Aono Scale		
Liu et al. (2017)^69^	Healthy male volunteers	Double‐blind randomized placebo‐controlled trial (Taylor Aggression Paradigm)	64 male adults (19–23 years)	MEQ	5 mg MLT	MLT increased the likelihood of selecting the highest punishment available
				TAP		
Malow et al. (2012)^66^	ASD	14‐week, uncontrolled, open‐label, before‐after dose‐escalation trial	24 children (3–9 years)	‐Child behavior checklist: Aggressive behavior	1–6 mg MTL	MLT had tendency to reduce aggressive behavior (*p* = .073)
				‐Repetitive behavior scale:		No effect of MLT on self‐injurious behavior
				Self‐injurious		
Mistraletti et al. (2015)^63^	Critically ill subjects	Double‐blind, placebo‐controlled randomized trial	82 adults requiring assisted ventilation	‐Anxiety VNR	6 mg MLT	MLT ↓ anxiety, agitation, and the amount of pharmacological sedation needed
				‐RASS		
Niederhofer (2012)^71^	ADHD	4‐week, open‐label, placebo‐controlled trial	10 (17–19 years)	Wender–Utah Questionnaire: fidgety subscale	25 mg agomelatine	Agomelatine ↓ fidgety compared to placebo
O'Neill et al. (2014)^72^	CRSD	Case report	61‐year‐old‐man	OAS‐MNR	25 mg agomelatine	Agomelatine ↓ challenging behavior
Özcengiz et al. (2011)^57^	Prevention of emergence agitation after general anesthesia with sevoflurane for esophageal dilation	Double‐blind, placebo‐controlled randomized trial	100 children (3–9 years)	EAS	0.1 mg/kg oral MTL	MTL ↓ postoperative agitation compared to placebo
Paavonen et al. (2003)^67^	ASPD	2‐week, uncontrolled, open‐label, before–after trial, and 3‐week follow‐up	15 children	CBCL	3 mg/day MLT	MLT appears associated with ↓ in aggressive behavior; no difference was detected in delinquent behavior
Pinkhasov et al. (2017)^73^	Elderly patients with delirium	Retrospective cohort study	125 elderly individuals	Antipsychotic use	Ramelteon (unspecified dose)	Ramelteon reduced the need to employ antipsychotics to manage agitation
Romero et al. (2021)^62^	Incidence of delirium among critically ill individuals	Retrospective cohort study	793 individuals with various underlying conditions	Incidence of delirium, RASS, use of antipsychotic, sedative, and opioid agents	Median doses:	No difference in the incidence rate of delirium between MLT, ramelteon, or no MLT groups. MLT group presented higher rates of agitation and sedation
					‐MLT 3 mg	
					‐Ramelteon 8 mg	
Samarkandi et al. (2005)^58^	Minor, elective surgery with general anesthesia	2‐week prospective, randomized, double‐blind, placebo‐controlled trial	105 children (2–5 years)	Pain/discomfort scale	0.1–0.25–0.5 mg/kg oral MTL	MTL ↓ post‐anesthetic excitement
					0.1–0.25–0.5 mg/kg oral MTL + acetaminophen	
Schroder et al. (2019)^68^	ASD, Smith‐Magenis syndrome	13‐week, double‐blind, placebo‐controlled randomized trial followed by a 91‐week open‐label trial	125 children (2–17.5 years)	SDQ	2–5 mg prolonged‐release MLT	MLT ↓ externalizing behaviors (hyperactivity‐inattention and conduct)
van der Heijden et al. (2007)^70^	ADHD	4‐week, double‐blind, placebo‐controlled randomized trial	105 children (6–12 years)	CBCL	3–6 mg MLT	No improvement in behavior was detected with MLT treatment
Wang et al. (2019)^64^	BPSD	Retrospective study	75 elderly individuals (80.7 years mean age)	NPI	12.5–50 mg agomelatine	Improvement in the delusion, hallucination, agitation/aggression, depression/dysphoria, anxiety, disinhibition, irritability/lability, motor disturbance, sleep/nighttime, and in behavior symptoms; no change for euphoria/elation symptoms
Yuge et al. (2020)^74^	NDD	26‐week, uncontrolled, open‐label, before–after dose‐escalation trial with a 2‐week follow‐up	99 children (10.4 years mean age)	ABC‐J	1, 2, or 4 mg melatonin (concomitant behavioral interventions)	Improvement in the irritability subscale at 26 weeks as compared with baseline

Abbreviations: ABC‐C, Aberrant Behavior Checklist—Community; ABC‐J, Aberrant Behavior Checklist—Japanese version; ABRS, Agitated Behavior Rating Scale; AD, Alzheimer's disease; ADHD, attention‐deficit/hyperactivity disorder; ASD, autism spectrum disorders; ASPD, Asperger disorder; BD, bipolar disorder; BEHAVE‐AD‐FW, Behavioral Pathology in Alzheimer's Disease Rating Scale; BPD, borderline personality disorder; BPSD, behavioral and psychological symptoms of dementia; CAM‐ICU, confusion assessment method‐ICU; CBCL, Children's Behavior Checklist for parents; CMAI, Cohen Mansfield Agitation Inventory; CRSD, circadian rhythm sleep disorders; EA, emergence agitation; EAS, emergence agitation scale; MDD, major depressive disorder; MEQ, Morningness Eveningness Questionnaire; MTL, melatonin; mYPAS, modified Yale Preoperative Anxiety Scale; NDD, neurodevelopmental disorders; NPI, Neuropsychiatry Inventory; OAS‐MNR, Overt Aggression Scale modified for neurorehabilitation; PAED, pediatric anesthesia emergence delirium; PHBQ, Post Hospitalization Behavior Questionnaire; RASS, Richmond Agitation‐Sedation Scale; SDQ, Strengths and Difficulties questionnaire; TAP, Taylor Aggression Paradigm; VNR, verbal numeric range.

Aggressive behavior is also one of the most prominent symptoms in children with autism.^65^ An open‐label study aimed at testing the possible therapeutic effects of MLT on sleep and behavioral symptoms in children with autism.^66^ MLT treatment (1–6 mg) appeared associated with a tendency (*p* = .073) to reduce aggressive behaviors, but with no noticeable effect on self‐injurious behaviors. A further open‐label study,^67^ reported on positive effects associated with MLT supplements in alleviating sleep and behavioral symptoms among 15 children affected by Asperger disorder. Most of the observed effects disappeared upon its discontinuation. Similarly, the results presented by Schroder et al.^68^ suggest that prolonged‐release MLT mini‐tablets at doses of 2–5 mg, may decrease aggression among children affected by an autism spectrum disorder or by Smith–Magenis syndrome. A randomized, double‐blind, placebo‐controlled trial involving male volunteers, reported a higher propensity for choosing to administer a high punishment to an opponent in the Taylor Aggression Paradigm with MLT exposure.^69^

In a randomized, placebo‐controlled trial, van der Heijden et al.^70^ evaluated the efficacy of 3–6 mg MLT in influencing sleep, behavior, quality of life, and cognitive performances among children affected by attention‐deficit/hyperactivity disorder (ADHD). The intervention improved sleep parameters, but no effect was observed in the remaining outcomes.^70^

Other studies tested whether nonselective MLT receptors ligands can reduce the severity of aggression. In an open‐label trial involving 10 children with ADHD, agomelatine appeared associated with reduced fidgeting and only slight sedative effects, suggesting that the observed decrease in irritability/agitation may be independent of the possible sedative effects of the drug.^71^ A case report described a reduction in challenging behaviors using agomelatine in a 61‐year‐old patient with severe brain damage due to a subarachnoid hemorrhage.^72^ Importantly, this effect was maintained during the entire 18 months follow‐up. Finally, a retrospective study of 125 patients with delirium tested whether ramelteon could decrease the need for an antipsychotic prescription for agitation.^73^ The authors found that ramelteon appeared associated with a lower incidence of antipsychotic use compared to controls.

### 3.3 Risk of bias of the included studies

**Figure 2:**
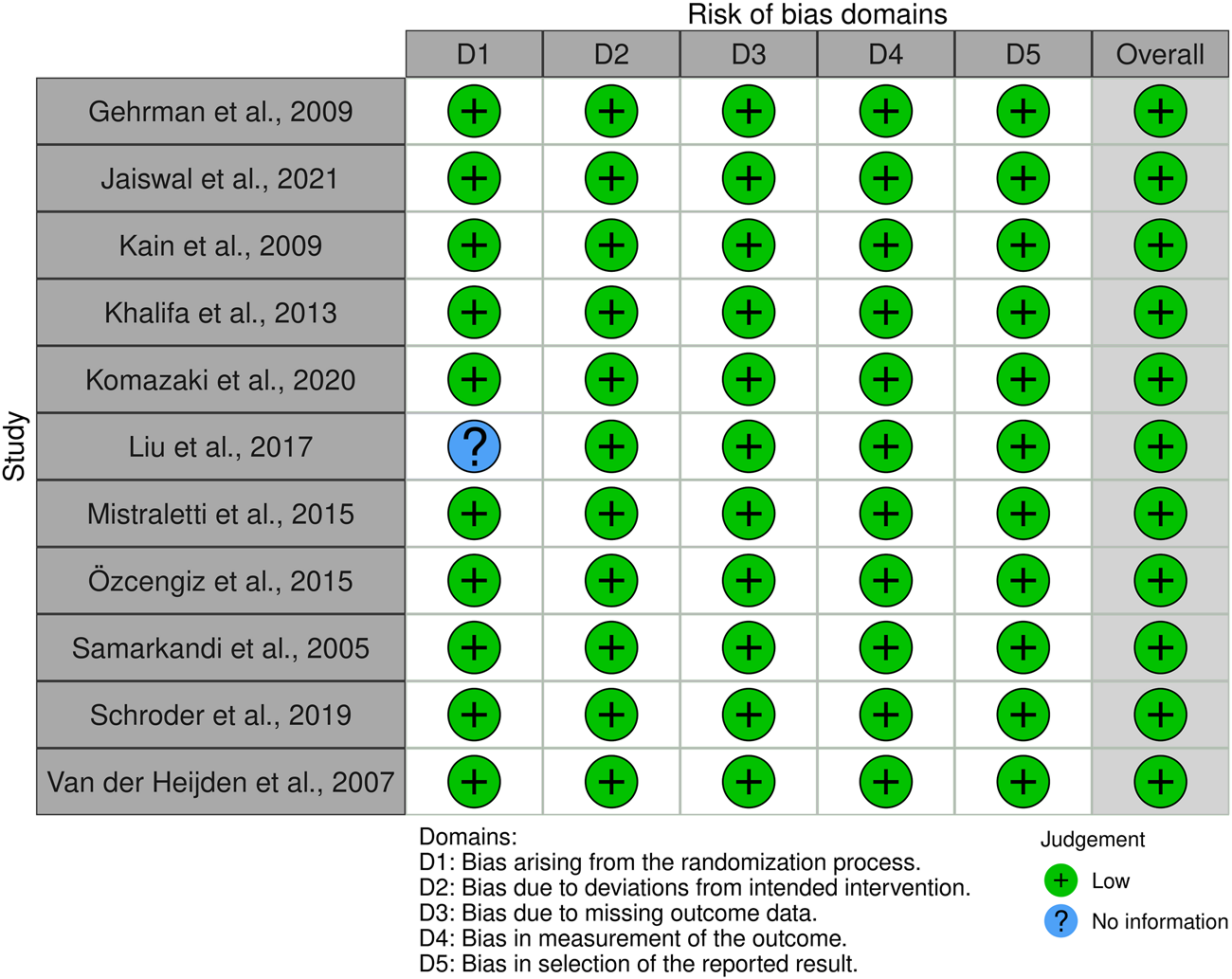
Illustration of the risk of bias for randomized clinical trials

**Figure 3:**
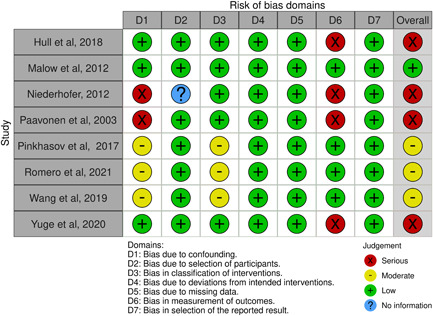
Illustration of the risk of bias for nonrandomized studies

The risk of bias for all the included randomized clinical trials was judged to be low, with one trial not specifying the employed randomization strategy.^69^ The results are summarized in Figure 2. A total of eight nonrandomized studies were eligible for the assessment according to the ROBINS‐I tool. One before–after study was judged at low risk of bias.^66^ The remaining three before–after trials were judged to be at serious risk of bias. One of them presented an inadequate assessment at baseline (i.e., a singular evaluation and no follow‐up time at baseline) and a high risk of measurement bias (i.e., unblinded raters evaluating subjective outcome scales).^67^ The remaining two presented a serious risk for possible measurement bias (i.e., subjective outcome measures evaluated by unblinded raters).^54^, ^74^ Three studies were judged at moderate risk for confounding by indication and for the assignment of intervention status due to the retrospective design.^62^, ^64^, ^73^ An open‐label, placebo‐controlled study was at serious risk of confoundings and measurement bias as no information was available on the total number of potentially eligible individuals.^71^ The results are summarized in Figure 3.

## 4 DISCUSSION

Our systematic review highlighted a series of important and novel findings. First, while a link between the photoperiod and thus variation in the physiological levels of MLT, and levels of aggressive behavior has been demonstrated in several studies in different species, there is still inadequate and limited evidence to draw any firm conclusion about a possible anti‐aggressive effect of MLT or MLT receptors agonists such as ramelteon or agomelatine. Most of the effects of MLT are mediated by its two receptors, but their possible selective role in aggressive/violent behaviors has not been explored yet. Growing evidence suggests that the two MLT receptors may control/modulate different physiopathological processes at central and peripheral levels.^4^, ^75^, ^76^, ^77^, ^78^, ^79^, ^80^, ^81^, ^82^ Importantly, selective MLT receptors agonists/partial agonists seem to be therapeutically superior to the nonselective MLT. For example, MT_2_ receptor partial agonists showed more potent hypnotic^77^, ^83^ and analgesic^80^, ^81^ effects than MLT. Given the link between the MLT system and aggressive behavior, it will be critical to understand if both MT_1_ and MT_2_ receptors or only one of the two subtypes are implicated, especially considering the possible development of new treatments for aggressive behaviors. Thanks to the recent advancement in medicinal chemistry and MLT ligands, we now have the availability of selective MT_1_ and MT_2_ agonists as well as antagonists. Thus, it will be possible to investigate the two MLT receptor subtypes' effects on aggressive behavior. In addition, research on aggressive behavior in MLT receptors knockout mice^84^, ^85^ as well as using selective or nonselective MT_1_ and MT_2_ antagonists could provide further insights. To date, we found only one preclinical study in California mice, which tested the nonselective MT_1_ and MT_2_ receptors antagonist luzindole finding no effect of the drug on aggressive behavior.^38^ However, only one single and likely very high dose (40 mg/kg) was tested.^38^ MLT receptors have been localized in brain regions^9^ intimately implicated in aggression including the prefrontal and frontal cortices, the hippocampus, the hypothalamus, and the raphe nuclei.^40^, ^86^ They modulate differently monoaminergic^75^, ^87^, ^88^ and glutamatergic^81^ neurotransmissions. Consequently, it is plausible to expect that MT_1_ and MT_2_ receptors may have a role in the pathophysiology of aggressive behavior.

Serotonin (5‐HT) is the most widely studied neurotransmitter in aggression.^22^, ^24^ It should be emphasized that 5‐HT is the precursor of MLT and thus variations in the levels of 5‐HT are likely to influence the levels of MLT. Although many studies have correlated cerebrospinal fluid levels of 5‐HT or its metabolites to aggression, no research has investigated possible variations in the levels of MLT. In addition, following a tryptophan depletion which lowers central 5‐HT levels, an increase in aggression has been observed.^89^, ^90^ But a tryptophan depletion also brings to a decrease in nocturnal MLT secretion.^91^ Therefore, it would be interesting also to differentiate the possible effects of MLT from that of 5‐HT in the increased aggression following the tryptophan depletion.

Preclinical studies in different rodents but also in fish have produced contrasting findings showing either increased, decreased, or no effect of MLT on aggression. We think that this apparently conflicting evidence may in part derive from the different experimental conditions employed, which may have strongly impacted the outcomes, as well as on the rodent model (nocturnal vs. diurnal). For example, studies differ significantly in the tested doses of MLT, the timing of the day of MLT administration, the time of the day, and the duration of the light/dark cycle in which the animals are tested. Sex‐specific effects are also worth further investigation for their possible clinical implications. Furthermore, several strains of mice produce no or low amounts of MLT due to defects in the activity of one or both the enzymes that sequentially transform 5‐HT into *N*‐acetylserotonin (the enzyme serotonin *N*‐acetyltransferase) and then *N*‐acetylserotonin into MLT (the enzyme hydroxyindole *O*‐methyltransferase). Mice of the C57BL/6 strain that have been largely used in the preclinical studies here reviewed are indeed among the MLT‐deficient inbred strains.^92^ As very few studies investigated the circulating levels of MLT, none examined the MLT concentrations in the brain at the time of testing, and the circadian variability in the levels of MLT has a strong implication on behavior and many other physiological functions (i.e., the expression of MLT receptors), the assessment of MLT levels in future studies may help further clarify these discrepancies. Finally, studies on the possible effects of MLT on aggression in nonmammalian vertebrates such as birds, reptiles, and amphibians are currently missing and need to be conducted to increase our understanding of the link between the MLT system and aggressive behavior.

In the literature here reviewed, we found discrepancies not only among preclinical studies but also between preclinical and clinical studies. For instance, while clinical studies highlighted a possible effect of MLT in reducing aggressive behavior, several preclinical studies in rodents reported the opposite. In this context, it is plausible that most of the observed discrepancies may rely on the difference in the circadian systems of these species (i.e., nocturnal vs. diurnal), rather than in the methodology. Indeed, virtually all rodents are nocturnal, meaning that high levels of MLT occur at times in which there is a peek in their behavioral activity, which includes also interactions with conspecifics and thus likely aggression. In contrast, in humans (Table 3) as well as in some of the fish studies (Table 2) here presented, the circadian system is primarily diurnal, meaning that the circulating levels of MLT are the highest when these animals are the least active. Moreover, given the link among MLT, circadian system, and sleep, and the fact that sleep dysfunctions can be a risk factor for aggressive behavior,^93^, ^94^ it can be hypothesized that the putative mechanism of MLT on aggressive behavior in humans relates to the quality of sleep, whereas in rodent models it is more likely related to serving a function during times of the day (or seasons) when heightened aggression is selected for. Future studies are warranted to examine this hypothesis as an example by looking at the link between the MLT system and aggression in diurnal animals (e.g., Meriones unguiculatus, Octodon degus, Arvicanthis niloticus, and Ammospermophilus leucurus)^95^ to see whether in these animals the findings more closely match with those found in human studies.

Among the clinical studies included in Table 3, those conducted in children/adolescents affected by different psychiatric disorders in which aggressive traits may be present, including oppositional defiant disorder, ADHD, conduct disorder, and autistic spectrum disorders, have shown a global agreement in highlighting positive effects of MLT or melatonergic drugs in controlling aggressive/violent behavior or traits associated with aggression such as irritability, anger, and agitation. Future randomized double‐blind placebo trials are thus welcome to support these encouraging findings especially because the prevalence of these developmental disorders is increasing worldwide, and up to now, there are no yet approved drugs to control aggressive‐like traits in children/adolescents. Of note, it should be emphasized that long‐term treatment with MLT seems to be generally well‐tolerated and safe.^30^, ^96^, ^97^ Studies also conducted in the elderly have suggested positive effects of MLT or melatonergic compounds alone or in combination with other drugs in controlling aggression, irritability, or agitation associated with different medical conditions. Several lines of evidence also suggest the efficacy of MLT use and its analog in preventing postoperative delirium, and these findings should also be further explored.^98^ All the included randomized controlled trials appear to be at low risk of bias, and therefore, despite the small sample sizes involved, the resulting evidence is deemed promising. However, evidence is still preliminary and more controlled trials are needed. The quality of the included nonrandomized trials is more heterogeneous, and thus the interpretation of their results appears more challenging.

In conclusion, although there are still many unanswered questions, this review reports current evidence on the neurobiological and psychopharmacology involvement of MLT and its receptors in aggressive/violent behavior, and given the encouraging findings, highlights the importance of conducting more studies in this still unexplored field of research.

## CONFLICTS OF INTEREST

The authors have no conflicts of interest to declare.

## AUTHOR CONTRIBUTIONS

Pasquale Paribello performed the literature search, contributed to the assessment of available evidence, and prepared the first draft of the manuscript. Mirko Manchia contributed to the literature search and the assessment of the available evidence and prepared the first draft of the manuscript. Marta Bosia, Federica Pinna, and Bernardo Carpiniello contributed to data collection and interpretation and critically revised the manuscript. Stefano Comai designed and oversaw the study, contributed to the literature search and the assessment of the available evidence, and prepared the first draft of the manuscript. All authors reviewed and approved the final version of this manuscript.

## Data Availability

Data sharing is not applicable to this article as no data sets were generated or analyzed during the current study.
